# Breast Cancer Screening Behaviors in Women Aged 40 Years and Over in a Semi-Urban Region in Turkey: Relationships with Health Beliefs

**DOI:** 10.3390/healthcare8020171

**Published:** 2020-06-13

**Authors:** Kevser Tarı Selçuk, Dilek Avcı, Gönül Yılmaz Dündar, Yeliz Mercan

**Affiliations:** 1 Department of Nutrition and Dietetics, Faculty of Health Sciences, Bandırma Onyedi Eylül University, Bandırma, 10200 Balıkesir, Turkey; 2 Nursing Department, Faculty of Health Sciences, Bandırma Onyedi Eylül University, Bandırma, 10200 Balıkesir, Turkey; davci@bandirma.edu.tr (D.A.); gyilmaz@bandirma.edu.tr (G.Y.D.); 3 Department of Health Management, Kırklareli University School of Health, 39000 Kırklareli, Turkey; yelizmercan@klu.edu.tr

**Keywords:** breast cancer, breast self-examination, mammography

## Abstract

In this study, we aimed to determine the breast cancer screening behavior of women and to investigate the relationship between health beliefs and screening behaviors. The study was cross-sectional. It was conducted between April 2017 and June 2017 with 416 women aged ≥40. The Sociodemographic Information Form and the Champion’s Health Belief Model Scale were used to collect data. In the statistical analysis, the number, percentage, mean, standard deviation, Pearson chi-square test, and multivariate binary logistic regression analysis were used. The rates for participating women performing breast self-examination, having clinical breast examination, and undergoing mammography were 11.8%, 8.9%, and 11.3%, respectively. Perceived susceptibility, seriousness, self-efficacy, benefits, health motivation, and perceived barriers were found to have strong associations with screening behaviors (*p* < 0.05). In this study, we found that few women performed breast self-examination, had clinical breast examination and mammography. In the present study, women perceived barriers related to both performing breast self-examination and undergoing mammography.

## 1. Introduction

Breast cancer (BC) is one of the most common types of cancer in women globally and accounts for a quarter of all new cancer cases, ranking second in cancer-related deaths across the world [1]. BC is the most fatal and most common type of cancer in women in Turkey, with an incidence of 47 per 100,000 [2].

The early diagnosis of BC is critical to reducing mortality. The literature shows that BC mortality rates have increased by about 14% since 2008 and that this increase is higher in developing countries due to delay in diagnosis and inadequate treatment [3,4]. In Turkey, in order to diagnose BC early and to decrease the BC-related mortality rate in women, the BC screening program has been carried out [5]. Within the scope of this program, the intention is to screen women in the 40–69 age group through mammography every two years; however, due to the shortage of specialized staff, problems with access to health service, and lack of awareness, the screening program currently covers only 20–30% of the target population in Turkey, and the majority of BC’s are diagnosed during the advanced stage [2]. In Turkey, the main screening method for early diagnosis of BC is mammography. The Ministry of Health recommends that women should undergo a mammography every two years from the age of 40, that clinical breast examination (CBE) should be performed in every woman participating in the screening in order to increase the effectiveness of mammography [5] and that the CBE should be repeated once a year [6]. Breast self-examination (BSE), which is still used as a screening method in developing countries, is not recommended by the World Health Organization (WHO) as a screening method, but is considered as an intervention that raises awareness among women at risk [7,8]. In randomized controlled studies performed recently, it has been shown that BSE is not an effective method for reducing cancer-related mortality [4]. However, in some other studies, it is stated that BSE may have contributed to the reduction of BC mortality by increasing women’s awareness of BC in some countries [9] and it is reported that BSE should be encouraged not as a method to reduce mortality but as a part of awareness-raising programs [9]. In Turkey, the Ministry of Health recommends that after the age of 20, every woman should perform BSE every month to raise their awareness of BC [5,6]. The low implementation rates of BSE (8.9–49.4%), of CBE (18.0–35.8%), and mammography (19.7–37.9%) in different regions of Turkey also reveal that to ensure positive change in BC screening behaviors, appropriate interventions should be planned as soon as possible [10,11,12,13,14,15].

Several factors may affect the performance of BC screening behaviors [16,17,18,19,20]. In the literature, it states that, in addition to sociodemographic and cultural characteristics, health beliefs related to the disease and its consequences affect health behaviors by increasing the motivation of individuals [21]. The Champion’s Health Belief Model is one of the models widely used to convey beliefs that can be effective in shaping health protection and health promotion behaviors [16,19,21]. In this model, women are encouraged to have BC screening tests to: find out whether they are at an increased risk of BC (perceived susceptibility); how severe the risk is considering its physical, mental, and social aspects (perceived severity); understand that early diagnosis would reduce negative health consequences (perceived benefits); understand that screening behavior is not costly, and that the person herself can perform the behavior (self-efficacy) [22]. Birhane et al. studied the rate of performing BSE, and found that it was high in women whose susceptibility and BSE benefit perception were high [23]; in the study by Eltvansy, the rate of having CBE was found to be high in women whose susceptibility, seriousness, and health motivation were high [24], and in the study by Shiryazdi et al., the rate of undergoing mammography was found to be high in women whose seriousness and mammography benefit perception were high [25]. In Turkey, studies investigated the BC screening behavior and health beliefs, although these studies mostly focused on one or two of the BC screening behaviors and were conducted with women of younger age [12,15,26,27]. In the recent studies conducted in Turkey, the rate of performing BSE was high in those whose perceptions of seriousness [15], self-efficacy [15,26,27], benefits [26,27], and motivation were high [26], and the rates of performing BSE [15,27] and undergoing mammography were high in those whose perception of barriers is low [15]. Investigating the three screening behaviors together in women aged 40 and older living in semi-urban areas to reveal the relationships between these behaviors and health beliefs will contribute to the determination of strategies that aim to increase BC screening behaviors in the region and to the planning of interventions. 

This study aimed to determine women’s BC screening behaviors and to investigate the relationship between health beliefs and screening behaviors.

## 2. Materials and Methods 

### 2.1. Study Type and Sample

This was a cross-sectional study. It was conducted in a semi-urban region of Bandirma, which is a district of the Balıkesir province, located on the coast of the Marmara Sea in the western part of Turkey.

To determine sample size, electronic databases were searched using keywords to access national studies on BC screening behaviors. In a recent national study, the rate of mammography was reported to be 19.0% in women (aged ≥30) [14]. The minimum sample was calculated to be 355 by taking *p* = 0.19, *α* = 0.05, and *d* = 0.03, using the Epi Info 7.2 program [28]. It was then decided to include 20% more people as substitutes and as a result, the target was to reach 426 women. The inclusion criteria were women who presented to the family health center (FHC) for any reason (*n* = 811) between 3 April 2017 and 28 June 2017 when the unit was not in mobile service, who had cognitively capable of answering the research questions (*n* = 808), and volunteered to participate in the study (*n* = 682). The exclusion criteria were then applied to the participants (*n* = 682), which was those who were under 40 years of age (*n* = 228), who were pregnant (*n* = 24), and who had a history of BC (*n* = 14), resulting in 416 women whose data were analyzed.

### 2.2. Data Collection Tools 

Data were collected by using the Sociodemographic Information Form and the Champion’s Health Belief Model Scale. The Champion’s Health Belief Model Scale was developed by Champion, and the validity and reliability study of the Turkish version of the scale was conducted by Gözüm. The five-point Likert-type scale consisted of 52 items and 8 subscales: susceptibility, seriousness, health motivation, BSE benefits, BSE barriers, BSE self-efficacy, mammography benefits, and mammography barriers. Internal consistency coefficients for the subscales were reported to range between 0.69 and 0.83 [21,29]. In this study, the Cronbach alfa coefficients of the subscales were calculated as 0.84–0.96.

### 2.3. Ethical Approval

Before data collection, ethics approval was received from the Balıkesir University Clinical Research Ethics Committee (Decision date and no: 2017/27). In this study, we followed the principles outlined in the Declaration of Helsinki for Human Studies.

### 2.4. Statistical Analysis 

The descriptive characteristics and BC screening behaviors of the participants were given in numbers and percentages. Pearson’s chi-square test was used to compare the screening behaviors of women in terms of their sociodemographic characteristics. Mean, standard deviation, and minimum and maximum values related to the subscale scores of the Champion’s Health Belief Model Scale were given. The relationship between health beliefs and BC screening behaviors was analyzed using the multivariate binary logistic regression analysis. For each regression model, BC screening behaviors were the dependent variables, and the subscales of the Champion’s Health belief Model scale were the primary independent variable, with age, education level, perceived economic status, and family history of BC added to the model as covariates. The Hosmer–Lemeshow goodness-of-fit test was used to determine how well the model fits with the data. The explanation of the model was evaluated with the Nagelkerke R square. All analyses were performed with SPSS Statistics for Windows version 23.0 (SPSS Inc., Chicago, IL, USA) and *p* < 0.05 was used to assess for significance.

## 3. Results

The sociodemographic characteristics of the participants are presented in Table 1.

The rates for women having performed BSE in the last month, having had CBE in the last year, and having undergone mammography in the last two years were 11.8%, 8.9%, and 11.3%, respectively (Table 2).

There was a statistically significant association between the BC screening behaviors and some sociodemographic characteristics (Table 3, *p* < 0.05). 

The mean scores obtained from participants for subscales are presented in Table 4.

According to the multivariate binary logistic regression analysis, the women who perceived more susceptibility, BSE self-efficacy, benefits of BSE, and fewer barriers to BSE were more likely to perform BSE. The women who perceived more seriousness and health motivation were more likely to have CBE. The women who perceived more seriousness, benefits of mammography, and fewer barriers to mammography were more likely to participate in mammography screening (Table 5, *p* < 0.05).

## 4. Discussion

In the present study, we determined that the rates for women performing BSE, having CBE, and undergoing mammography were approximately 12%, 9%, and 11%, respectively. It was reported in the literature that the rates for women performing BSE, having CBE, and undergoing mammography in developing countries range from 17% to 50%, from 20% to 47%, and from 20% to 55%, respectively [30,31,32,33,34,35]. In studies carried out at a local level in Turkey, the rates for women performing BSE, having CBE, and undergoing mammography were reported to range from 13% to 49%, from 15% to 36%, and from 20% to 38%, respectively [10,11,12,13,14,36,37]. In the present study, we studied the monthly BSE, annual CBE, and biannual mammography rates in line with the recommendation of the Turkish Ministry of Health, and found that BSE, CBE, and mammography rates were lower than those in other studies carried out in Turkey and other developing countries [2,10,11,12,13,14,36,37,38]. This difference may be due to differences in the methods used to investigate BC screening behaviors and from the differences between the participants’ age groups in these studies [12,13,14,15,30,31,33].

In this study, we determined that the most significant relationships were between health beliefs and BSE (in the last month), CBE (in the last year), and mammography (in the last two years), after adjusting for age, educational level, family history, and perceived economic status among adults. In the present study, women who performed BSE had a higher perception of their susceptibility [39,40] and of their self-efficacy [10,18,26,27], which reflected two of the subscales of the Champion’s Health Belief Model. This is consistent with the results in the literature. Susceptibility is a person’s beliefs regarding the possibility of facing a health threat. Self-efficacy is defined as “a person’s belief that he/she has the competence, determination, and commitment to perform the actions needed to achieve a desired result” [16,40,41]. In this context, it can be said that a woman’s belief in the possibility of her developing BC and in her ability to take action against the possibility of developing BC are strong determinants in the acquisition of cancer prevention behaviors. 

In this study, we found that the women who had a high perception of seriousness underwent CBE and mammography more frequently. The concept of perceived seriousness is defined as “a person’s beliefs about the severity of the disease”. Perceived seriousness includes the assessment of the possible consequences of the illness, such as death, disability, pain, and social losses [21,40]. Studies have indicated that women who perceive the seriousness of BC, and who consider that they are at risk of developing BC, tend to undergo CBE and mammography more frequently [30,42]. In this study, we also found that women whose health motivation was high had CBE more often, where health motivation is defined by “their willingness to display a behavior to protect their health”. This result is consistent with other results in the literature [30,43]. These results are consistent with the theoretical structure of the Health Belief Model and can be interpreted as follows: the perception of seriousness increases motivation and encourages women to display positive health behaviors, such as undergoing CBE and mammography. 

The perceived benefit is the extent to which the person perceives the change in behavior as beneficial and the extent to which he/she believes he/she can prevent the risk of illness if he/she makes the required behavioral change [21,44]. The literature shows that high levels of perceived benefits regarding BC contribute to an early diagnosis of the disease and that this increases treatment possibilities, a reduction in cancer-related mortality, prolonged survival, and increased quality of life [45]. In the present study, the BSE and mammography benefit perception was high in women who performed BSE and underwent mammography. The literature states that there is a positive association between perceived benefits, the performing of BSE, and the undergoing of mammography [10,26,31]. 

On the other hand, this study found that the likelihood of performing BSE and undergoing mammography decreased in women whose perceptions of the barriers to BSE and mammography were high. A perceived barrier is the perception of factors that impede a health-related protective behavior or that make it difficult to display such a behavior [21,40]. These results were consistent with the literature [18,19,45,46], and it can be said that a high perception of the benefit and a low perception of the barriers play important roles in the regular achievement of screening behaviors.

In this study, three screening behaviors for the early diagnosis of BC were evaluated and the evaluation of whether these screening behaviors are performed at the recommended frequencies is one of the strengths of the study; however, this study has some limitations. One of these limitations is that the study had a cross-sectional nature and thus the causal relationship was not displayed. Other limitations of the study were that the questioning of the participants’ BC screening behaviors was based on self-reporting, that the study was conducted with women who presented to only one FHC, and that the results could not be generalized to the wider society. Another limitation was that because the current version of the Champion’s Health Belief Model Scale [47] was not adapted to Turkish, an older version had to be used.

## 5. Conclusions

In this study, the rates for participants performing BSE, undertaking CBE, and undergoing mammography ranged between 9% and 12%. Given that the participants in the present study group consisted of women aged 40 and over, who are in an at-risk group for BC, it can be said that these rates were remarkably low. Positive attitudes toward perceived susceptibility, seriousness, self-efficacy, benefits, and health motivation have strong associations with screening behaviors. 

In the literature, it is stated that the most important barrier in developing a behavior is the perception of a barrier, and that this perception can be changed by education, counseling, and attempts aimed at increasing access to health services, and thus, as the perception of a barrier decreases, benefit perception increases [19]. In the present study, the perception of a barrier was related to both performing BSE and undergoing mammography. 

Health professionals working in primary healthcare institutions should aim to increase women’s knowledge and awareness of breast cancer and screening methods, inform them about the national breast cancer screening program, and encourage them to benefit from it. Women should be encouraged to perform and improve their BSE skills. In a future study, focus groups or in-depth interviews should be conducted to reveal the causes of the perception of barriers towards performing BSE and undergoing mammography, and appropriate initiatives should be planned to eliminate the causes identified. Education and consultancy programs that focus on increasing motivation, susceptibility, seriousness, and understanding of the benefits of screening behaviors while eliminating barriers should be organized by primary healthcare institutions. To assess BC screening behaviors and investigate their relationship between BC screening behaviors and health beliefs in women, community-based studies should be conducted. In future studies, we should not only investigate factors related to health beliefs but also investigate psychosocial factors and factors related to the use of health services that may be associated with screening. We suggest that BC screening behaviors should be evaluated, not based on self-reporting, but instead based on the records of health institutions. 

## Figures and Tables

**Table 1:** Sociodemographic characteristics of the participants (*n* = 416).

Sociodemographic Characteristics	*n*	%
Age		
40–49	157	37.7
50–59	119	28.6
≥60	140	33.7
Marital status		
Married	333	80.0
Single/divorced/widow	83	20.0
Education level		
Primary school or lower	225	54.1
Secondary school	158	38.0
High school and higher	33	7.9
Employment status		
Yes	46	11.1
No	370	88.9
Perceived economic status		
Good	52	12.5
Moderate	305	73.3
Bad	59	14.2
Health insurance		
Yes	403	96.9
No	13	3.1
Family history		
Yes	54	13.0
No	362	87.0

**Table 2:** Breast cancer screening behaviors of the participants (*n* = 416).

Screening Behaviors	*n*	%
Breast self-examination (BSE) *		
Yes	49	11.8
No	367	88.2
Clinical breast examination (CBE) **		
Yes	37	8.9
No	379	91.1
Mammography ***		
Yes	47	11.3
No	369	88.7

* In the last month. ** In the last year. *** In the last two years.

**Table 3:** Breast cancer screening behaviors in the participants according to sociodemographic characteristics.

Sociodemographic Characteristics	BSE			CBE			Mammography		
	Yes	No	*p* *	Yes	No	*p* *	Yes	No	*p* *
	%	%		%	%		%	%	
Age									
40–49	14.0	86.0	0.021	8.3	91.7	0.943	10.8	89.2	0.436
50–59	16.0	84.0		9.2	90.8		14.3	85.7	
≥60	5.7	94.3		9.3	90.7		9.3	90.7	
Marital status									
Married	14.1	85.9	0.003	7.5	92.5	0.047	11.4	88.6	0.884
Single/divorced/widow	2.4	97.6		14.5	85.5		10.8	89.2	
Education level									
Primary school or lower	8.4	91.6	0.041	8.9	91.1	0.024	8.0	92.0	0.000
Secondary school	14.6	85.4		6.3	93.7		10.1	89.9	
High school and higher	21.2	78.8		21.2	78.8		39.4	60.6	
Employment status									
Yes	17.4	82.6	0.211	6.5	93.5	0.549	17.4	82.6	0.166
No	11.1	88.9		9.2	90.8		10.5	89.5	
Perceived economic status									
Good	5.8	94.2	0.290	19.2	80.8	0.019	19.2	80.8	0.031
Moderate	13.1	86.9		7.2	92.8		11.5	88.5	
Bad	10.2	89.8		8.5	91.5		3.4	96.6	
Health insurance									
Yes	11.7	88.3	0.682	8.9	91.1	0.877	10.9	89.1	0.173
No	15.4	84.6		7.7	92.3		23.1	76.9	
Family history of BC									
Yes	16.7	83.3	0.232	7.4	92.6	0.681	27.8	72.2	0.000
No	11.0	89.0		9.1	90.9		8.8	91.2	

* Pearson chi-square test.

**Table 4:** Mean scores on the Health Belief Model Subscale.

Health Belief Model Subscale	$\bar{X}$ Standard Deviation (SD)	Min.–Max.	Score Range
Susceptibility	8.41 ± 2.26	3.00–15.00	3.00–15.00
Seriousness	20.34 ± 5.01	6.00–30.00	6.00–30.00
Health motivation	19.03 ± 3.85	5.00–25.00	5.00–25.00
BSE benefits	14.17 ± 3.17	4.00–20.00	4.00–20.00
BSE barriers	22.96 ± 7.46	8.00–37.00	8.00–40.00
BSE self-efficacy	30.33 ± 9.39	10.00–50.00	10.00–50.00
Mammography benefits	18.21 ± 4.34	5.00–25.00	5.00–25.00
Mammography barriers	33.99 ± 10.66	11.00–55.00	11.00–55.00

**Table 5:** The relationship between the participants’ health beliefs and screening behaviors according to multivariate binary logistic regression analysis.

Variables	BSE		CBE		Mammography	
	UOR (%95 CI)	AOR (%95 CI)	UOR (%95 CI)	AOR (%95 CI)	UOR (%95 CI)	AOR (%95 CI)
Susceptibility	1.27 (1.14–1.41) ***	2.02 (1.02–3.99) *	0.99 (0.88–1.12)	0.88 (0.77–1.02)	1.10 (0.99–1.22)	1.14 (0.98–1.33)
Seriousness	1.51 (1.33–1.71) ***	1.39 (0.93–2.07)	1.12 (1.04–1.20) **	1.08 (1.02–1.17) *	1.1 (1.04–1.19) **	1.15 (1.07–1.24) ***
Health motivation	1.01 (0.93–1.08)	0.98 (0.76–1.26)	1.45 (1.26–1.68) ***	1.42 (1.21–1.66) ***	1.08 (1.01–1.16) *	0.93 (0.85–1.02)
BSE benefits	2.66 (2.13–3.33) ***	2.43 (1.37–4.29) **	NA	NA	NA	NA
BSE barriers	0.65 (0.58–0.73) ***	0.67 (0.53–0.84) ***	NA	NA	NA	NA
BSE self-efficacy	1.39 (1.28–1.51) ***	1.55 (1.11–2.17) **	NA	NA	NA	NA
Mammography benefits	NA	NA	NA	NA	1.22 (1.12–1.33) ***	1.45 (1.27–1.65) ***
Mammography barriers	NA	NA	NA	NA	0.95 (0.92–0.98) **	0.90 (0.87–0.94) ***

UOR: Unadjusted Odds Ratio. AOR: Adjusted Odds Ratio. CI: Confidence Interval. NA: Not applicable. * *p* < 0.05, ** *p* < 0.01, *** *p* < 0.001. Odds ratio adjusted for age, education level (primary school or lower: 0, secondary school: 1, high school and higher: 2), perceived economic status (bad: 0, moderate: 1, good: 2), family history of BC (no: 0, yes: 1). BSE: Hosmer and Lemeshow test: chi square = 0.844, *p* = 0.998, Nagelkerke R square = 0.941, CBE: Hosmer and Lemeshow test: chi square = 13.360, *p* = 0.100, Nagelkerke R square = 0.247, Mammography: Hosmer and Lemeshow test: chi square = 2.814, *p* = 0.902, Nagelkerke R square = 0.493.
